# A Case of Ochrobactrum intermedium Bacteremia Secondary to Cholangitis With a Literature Review

**DOI:** 10.7759/cureus.14648

**Published:** 2021-04-23

**Authors:** Ihab Kassab, Nadine Sarsam, Saif Affas, Mohamad Ayas, Ji H Baang

**Affiliations:** 1 Internal Medicine, University of Michigan, Ann Arbor, USA; 2 Accident and Emergency Services, Luton & Dunstable University Hospital, Luton, GBR; 3 Internal Medicine, Ascension St. John Hospital, Detroit, USA; 4 Internal Medicine/Infectious Disease, University of Michigan, Ann Arbor, USA

**Keywords:** ochrobactrum species, ochrobactrum intermedium, cholangitis, ascending cholangitis, primary sclerosing cholangitis, bacteremia

## Abstract

*Ochrobactrum* species are gram-negative, non-lactose fermenting, aerobic bacilli closely related to *Brucella* genus. *Ochrobactrum intermedium *(*O. intermedium*) is an emergent human pathogen that is difficult to differentiate from other *Ochrobactrum* species by conventional methods. It is known to infect immunocompromised hosts, has the propensity for abscess formation, and is known for its multidrug resistance. We describe the case of an 84-year-old woman with a background of primary sclerosing cholangitis who presented with fatigue, fever, and syncope. Blood cultures grew *O. intermedium*. Magnetic resonance cholangiopancreatography and endoscopic retrograde cholangiopancreatography were consistent with cholangitis. Cultures from the biliary duct confirmed the same microorganism. The patient was successfully treated with minocycline. Although rare, *O. intermedium* should be considered as a differential diagnosis in patients with biliary and gut pathology, particularly in immunocompromised patients.

## Introduction

*Ochrobactrum* species are gram-negative, non-lactose fermenting, aerobic bacilli closely related to the *Brucella* genus. The *Ochrobactrum* genus was first identified in 1988. It was previously categorized as the Centers for Disease Control and Prevention (CDC) group Vd 1-2 [1-4]*. Ochrobactrum* species can be found in the environment such as water, soil, animals, and plants, as well as in polluted environments [1,5]. It is also thought to be part of the normal flora of our large intestines [6]. The *Ochrobactrum* genus includes nine different species: *O. anthropi*, *O. intermedium*, *O. lupini*, *O. tritici*, *O. grignonense*, *O. gallinifaecis*, *O. oryzae*, *O. pseudintermedium*, and *O. cytisi*. Out of these, only *O. anthropi*, *O. intermedium*, and *O. pseudintermedium* have been reported to cause clinical manifestations [7].

Although *Ochrobactrum* species have been recognized as opportunistic human pathogens with low virulence, most of the cases reported are about *O. anthropi*, which is usually associated with intravenous catheter infections and is commonly seen in immunocompromised patients [8,9]. On the other hand, *O. intermedium*, which is an emergent human pathogen that is difficult to differentiate from other *Ochrobactrum* species by conventional methods, has been rarely reported in the literature. *Ochrobactrum intermedium* has the tendency to form microabscess and is notorious for its multidrug resistance [5].

We present a case of cholangitis secondary to *O. intermedium* infection in an 84-year-old patient who was successfully treated with minocycline.

## Case presentation

**Figure 1:**
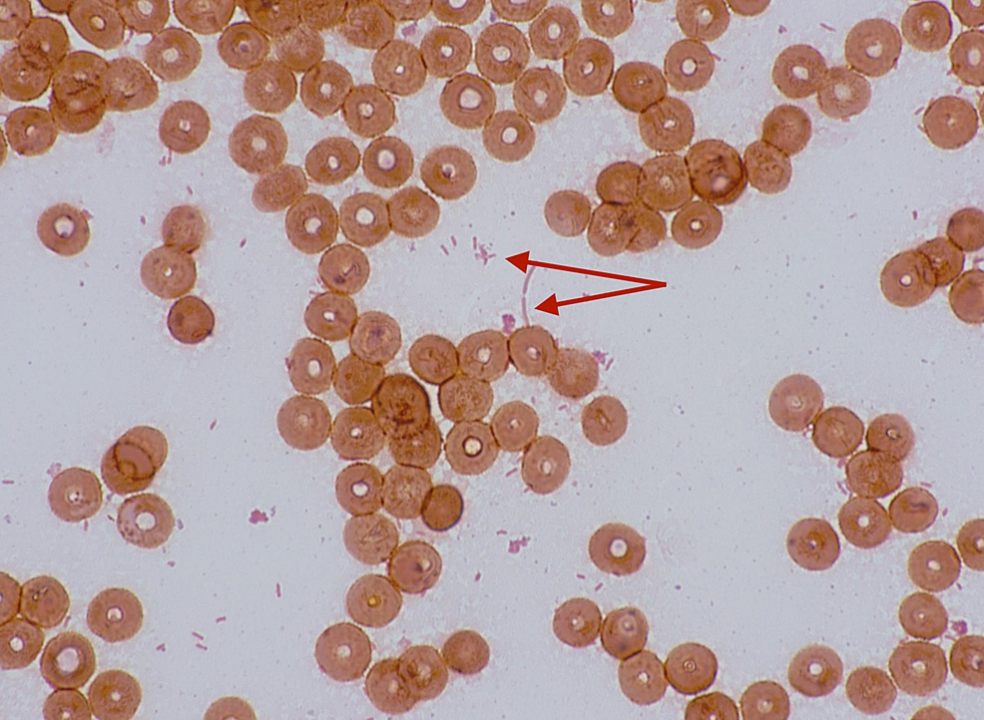
Gram staining showing gram-negative rods (red arrows)

An 84-year-old woman with a history of primary sclerosing cholangitis (PSC) with compensated cirrhosis without ascites, esophageal varices, or encephalopathy presented to our hospital with fatigue, fever, and syncope. Review of systems was negative for abdominal pain, cough, shortness of breath, and urinary symptoms. She had no history of weight loss. She denied any use of tobacco, alcohol, or illicit drugs. She had a body mass index (BMI) of 22 kg/m^2^. She was noted to have a fever of 39.5°C with a normal pulse and blood pressure. On examination, she was awake and oriented, with no focal neurological signs. She had normal breath and heart sounds on auscultation. Her abdomen was soft and nontender. Laboratory work showed a white blood cell count of 12.7 K/uL (4-10 K/uL) and a hemoglobin of 14.7 g/dL (12-16 g/dL). Her comprehensive metabolic panel was noted for elevated aspartate aminotransferase of 68 IU/L (8-30 IU/L), alanine aminotransferase of 84 IU/L (< 35 IU/L), alkaline phosphatase of 200 IU/L (40-116 IU/L), and total bilirubin of 1.5 mg/dL (0.2-1.2 mg/dL). The patient's baseline liver enzymes were normal. A chest X-ray was performed, which revealed right middle lobe atelectasis that appeared unchanged from prior chest X-rays. Blood cultures were obtained, and the patient was started on empirical intravenous antibiotics including ampicillin/sulbactam 3 g and azithromycin 500 mg. Gram staining revealed gram-negative rods (Figure 1).

Blood cultures grew *O. intermedium *within 48 hours of collection that was speciated with the matrix-assisted laser desorption/ionization-time of flight mass spectrometry (MALDI-TOF MS) method.

Antibiotics were broadened to meropenem while awaiting sensitivity. Despite that, the blood cultures remained positive for the same microorganism for three consecutive sets.

**Figure 2:**
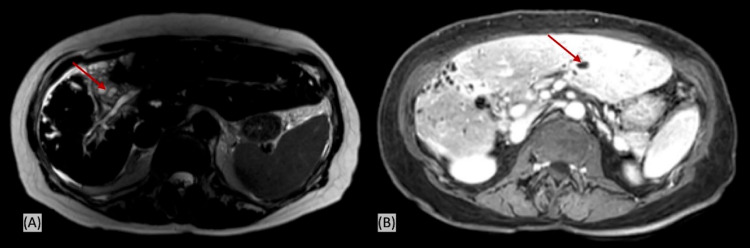
Magnetic resonance cholangiopancreatography (MRCP) showing diffuse common bile duct stricturing and biliary duct dilatation Panel A: T2-weighed image; panel B: post-contrast T1-weighted image with fat saturation

We performed a transthoracic echocardiography, which ruled out endocarditis. Additionally, magnetic resonance cholangiopancreatography (MRCP) was performed, which showed diffuse stricturing of the common bile duct throughout its course. There was severe multifocal stricturing of the central intrahepatic biliary tree with peripheral bile duct dilatation and irregularity compatible with sclerosing cholangitis. The MRCP imaging showed slight global worsening in the degree of biliary dilatation from prior scans. There was no evidence of hepatocellular carcinoma (Figure 2).

**Figure 3:**
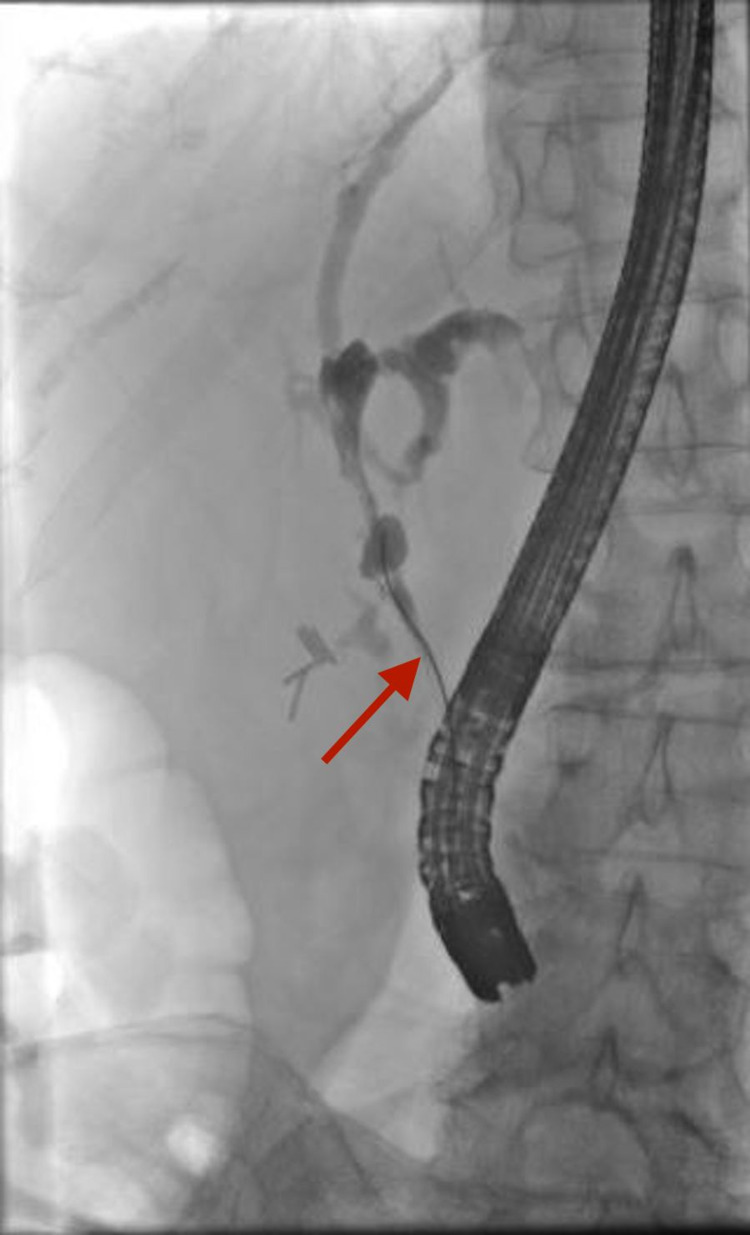
Endoscopic retrograde cholangiopancreatography (ERCP) showing a severe extrahepatic bile duct stricture (red arrow) with left filing defects due to sludge

This was followed by endoscopic retrograde cholangiopancreatography (ERCP), which showed a single severe biliary stricture in the extrahepatic bile duct resulting in intrahepatic ductal dilatation. A 6-mm biliary sphincterotomy was made. The biliary tree was swept revealing sludge and pus. The common bile duct was dilated with improved drainage after dilation. Cells for cytology were obtained by brushing into the entire extrahepatic bile duct (Figure 3).

Common bile duct brush cytology showed no malignant cells. CA19-9 was negative. The patient was switched to minocycline as per sensitivity and was discharged home to finish a course of 14 days of minocycline.

## Discussion

*O. intermedium* represents an emerging species that was first separated from *O. anthropi* in 1998 as it was not possible to differentiate them by standard tests until then. Additionally, *O. intermedium* has a closer relation to the *Brucella* genus than other species of *Ochrobactrum* as it shares 98.8% of its 16S rRNA with this genus for which sophisticated molecular tests are required for differentiation, such as 16S rDNA gene sequencing, recA-PCR RFLP (Restriction Fragment Length Polymorphism), and MALDI-TOF MS method. The 16S rDNA gene sequencing is considered the gold standard technique in differentiating between those species. However, due to its high cost and lack of availability, MALDI-TOF is used alternatively, which has shown to be an effective and reliable tool in identifying microorganisms [1,3,4,7,10]. Our specimen was speciated with the MALDI-TOF MS. 

Only a few cases of *O. intermedium* infections have been described in the literature. We conducted a retrospective PubMed literature search and found a total of 7 reported cases of *O. intermedium* (Table 1).

**Table 1:** Retrospective PubMed literature search for reported Ochrobactrum intermedium cases NR, not reported

Author (Year)	Age	Gender	Immunity Status	Presentation	Treatment
Möller et al., 1999 [6]	45	Female	Compromised	Patient presented with sepsis a month after receiving a liver transplant due to primary sclerosis cholangitis and was found to have cholangitis as well as multiple micro-abscesses in the liver and one large abscess in the dome of the right lobe	Imipenem, tobramycin
Apisarnthanarak et al., 2005 [9]	74	Male	Compromised	Patient with a bladder cancer presented with a large bowel obstruction, requiring a colostomy a month after radical cystectomy	Imipenem, ciprofloxacin
Vaidya et al., 2005 [11]	49	Male	Competent	Patient presented with lower abdominal pain and appendicitis complicated by perirectal abscess followed by bowel obstruction and pelvic abscess	Fluoroquinolones
Dharne et al., 2008 [12]	NR	NR	NR	Incidental finding in an asymptomatic patient on stomach biopsy	NR
Jacobs et al., 2013 [13]	34	Male	Competent	Patient presented with endophthalmitis secondary to a metallic intraocular foreign body	Intravitreal, moxifloxacin
Bharucha et al., 2017 [14]	23	Male	Compromised	Patient on hemodialysis via right internal jugular catheter after failing of renal transplant but still on immunosuppressor presented with endocarditis	Meropenem
Hirai et al., 2016 [10]	86	Male	Competent	Patient who had a stroke and pontine infarction developed a peripheral venous catheter infection	Meropenem

In the reported cases, the median age of infection with *O. intermedium* was 47 years (range: 23-86 years) [10,14]. Five cases were males [9-11,13,14], one case was a female [6], and in one case the gender was not reported. *Ochrobactrum* intermedium infection was largely associated with bacteremia and systemic manifestations [6,9-11,14]. Out of the seven reported cases, six patients had bacteremia, three of whom were immunocompromised [6,9,14]. Five of the reported cases were associated with systemic manifestations, as seen in our patient. Two cases were not associated with any systemic disease, one of which was a case of endophthalmitis secondary to metallic intraocular foreign body injury [13], and the other was an incidental finding of the microorganism on a stomach biopsy performed during esophagogastroduodenoscopy (EGD) for a patient with non-ulcerative dyspepsia [12]. Two cases were associated with intestinal obstruction as a complication [9,11]. Abscess formation was described in three cases [6,9,11].

One case had a history of PSC associated with advanced liver cirrhosis, who presented with signs of septicemia one month after orthotopic liver transplantation and was diagnosed with cholangitis secondary to *O. intermedium*. The patient later developed ischemic-type biliary lesions that required re-transplantation six weeks post primary transplantation [6]. Our case is the second reported case of cholangitis secondary to *O. intermedium*. Similar to the aforementioned case, our patient had a history of PSC and developed cholangitis secondary to *O. intermedium*. However, our patient had early stages of cirrhosis (Child-Pugh A5) without complications and had no history of organ transplant. Although her liver cirrhosis was mild, it may have subjected her to a form of depressed immunity as studies have shown that the liver is a primary surveillance organ for intravascular infection and plays an important role in filtering various fungal and bacterial pathogens, and, therefore, patients with liver dysfunction are at increased risk of infection from a variety of pathogens [15].

Two cases were treated with fluoroquinolones [11,13] and one case with fluoroquinolones in combination with imipenem [9]. Meropenem alone was used in two cases [10,11], while imipenem and tobramycin were used in one case [6].

*Ochrobactrum intermedium* microorganism is capable of producing pyogenic infections and has the characteristic of multidrug resistance. It is resistant to multiple families of antibiotics such as ß-lactum including penicillins, cephalosporins, and sometimes carbapenems. This makes treatment of *Ochrobactrum* bacteria often challenging, with most strains susceptible to fluoroquinolones, aminoglycosides, carbapenems, and trimethoprim-sulfamethoxazole (TMP-SMX) [4,6,16-19].

Our patient was sensitive to TMP-SMX, fluoroquinolones (ciprofloxacin and levofloxacin), tetracyclines (doxycycline and minocycline), carbapenems (imipenem and meropenem), and amikacin, and resistant to piperacillin/tazobactam, tobramycin, gentamicin, aztreonam, and ceftazidime (Table 2).

**Table 2:** Antibiotics susceptibility table for Ochrobactrum intermedium MIC, minimum inhibitory concentration

Antibiotics	Susceptibility Profile	MIC
Amikacin	Sensitive	16 mcg/mL
Aztreonam	Resistant	>16 mcg/mL
Cefepime	Intermediate	16 mcg/mL
Ceftazidime	Resistant	>16 mcg/mL
Ciprofloxacin	Sensitive	0.25 mcg/mL
Doxycycline	Sensitive	≤2 mcg/mL
Gentamicin	Resistant	>8 mcg/mL
Imipenem	Sensitive	≤1 mcg/mL
Levofloxacin	Sensitive	≤0.5 mcg/mL
Meropenem	Sensitive	≤1 mcg/mL
Minocycline	Sensitive	≤2 mcg/mL
Piperacillin/tazobactam	Resistant	>64 mcg/mL
Tobramycin	Resistant	>8 mcg/mL
Trimethoprim/sulfamethoxazole	Sensitive	≤2 mcg/mL

## Conclusions

We described a rare case of *O. intermedium *ascending cholangitis complicated by bacteremia. Although rare, this organism should be considered as a pathogen in patients with biliary and gut pathology. The similarity of this bacterium with other *Ochrobactrum* species and with the *Brucella *genus makes the diagnosis challenging, requiring sophisticated methods for differentiation. We reported this case to heighten physicians’ awareness on this type of bacteria, including early detection, identification of the source of infection, and the use of appropriate antibiotics for treatment. *Ochrobactrum *intermedium should be considered as a differential diagnosis particularly in immunocompromised patients presenting with cholangitis, abscess formation, endocarditis, and catheter-associated infections.
